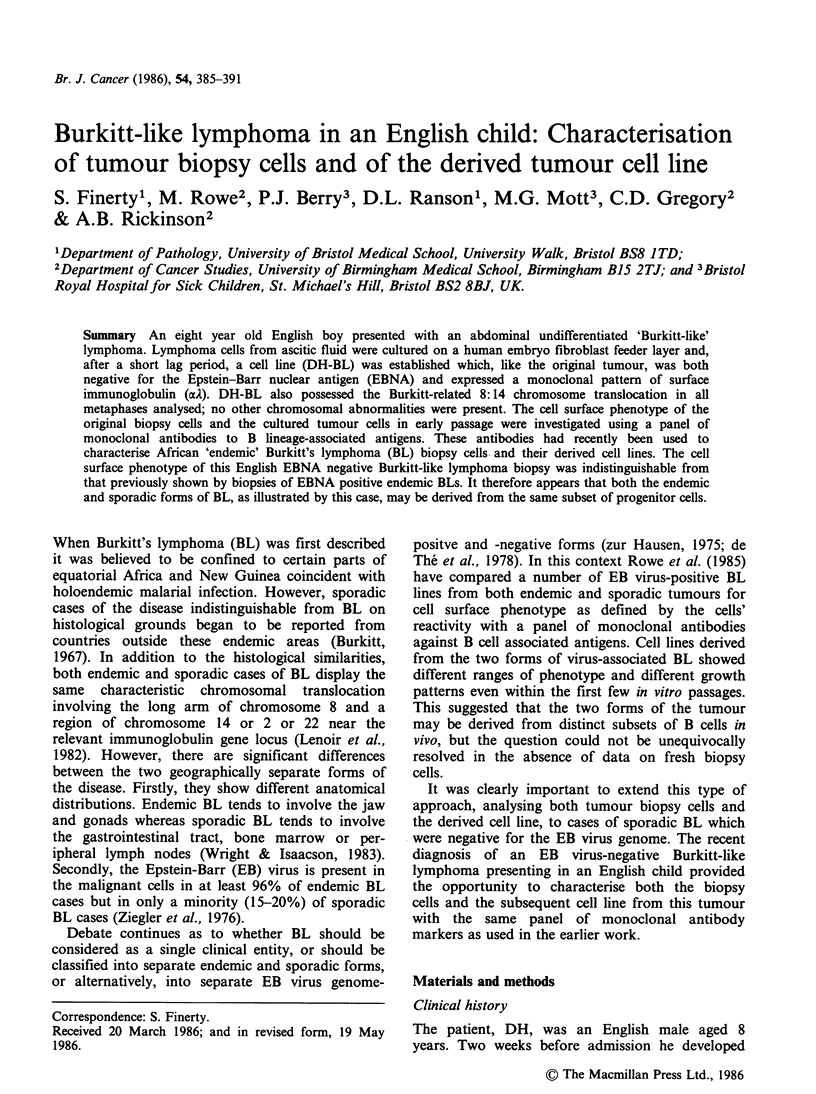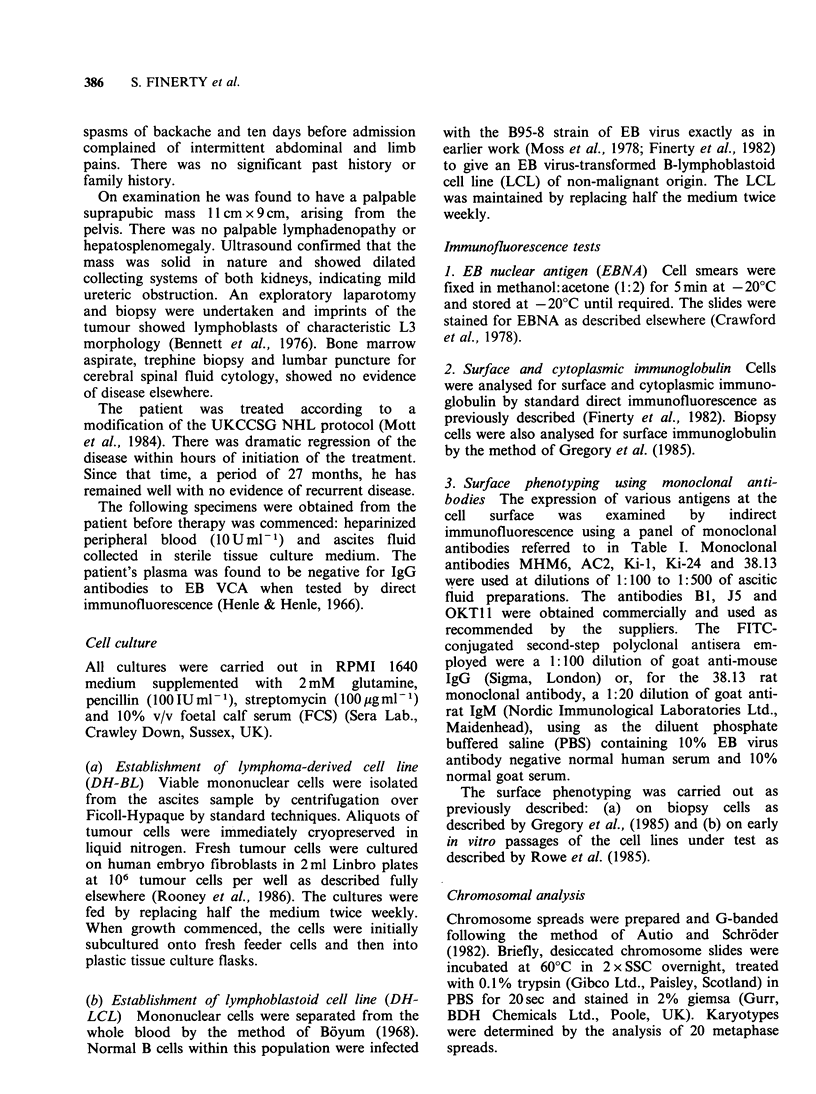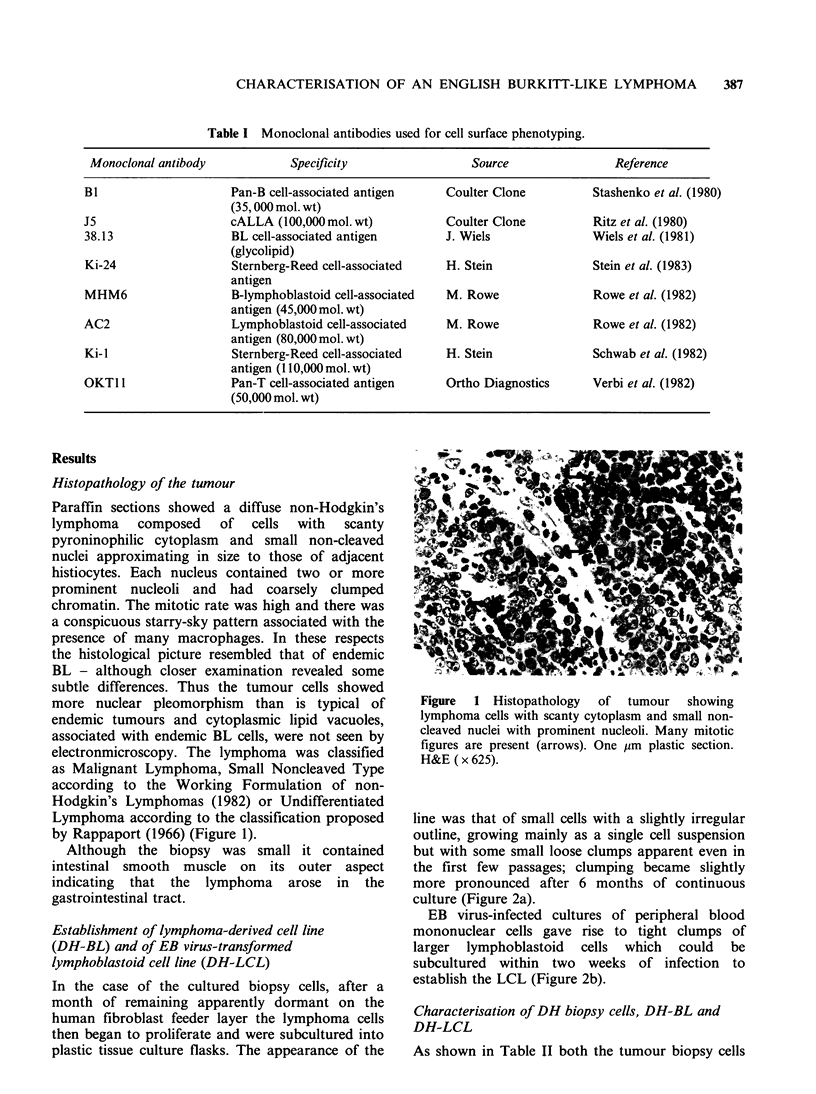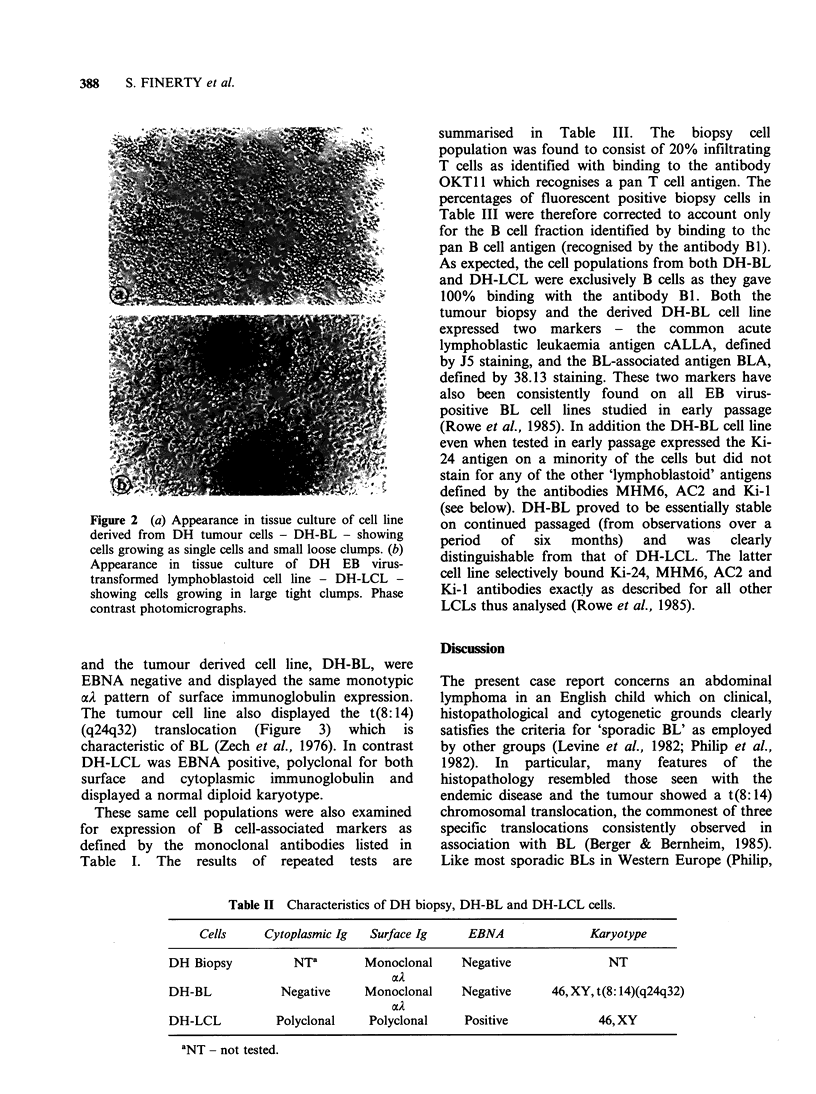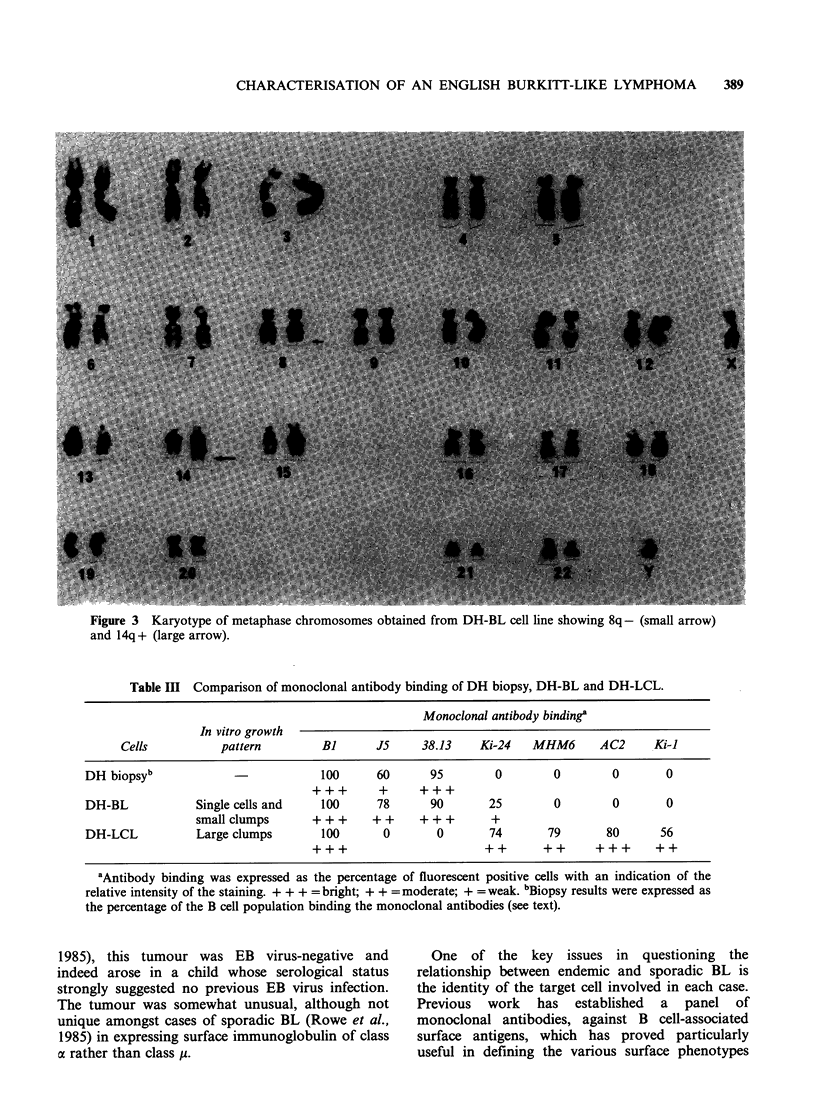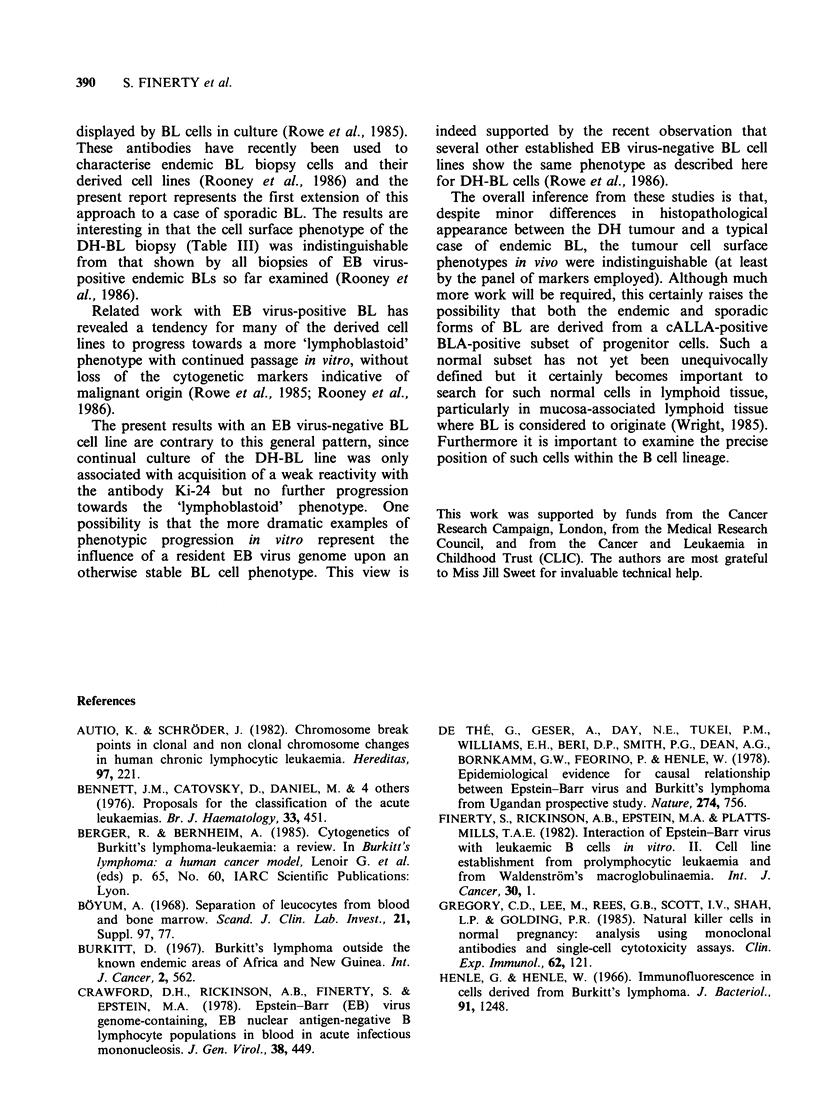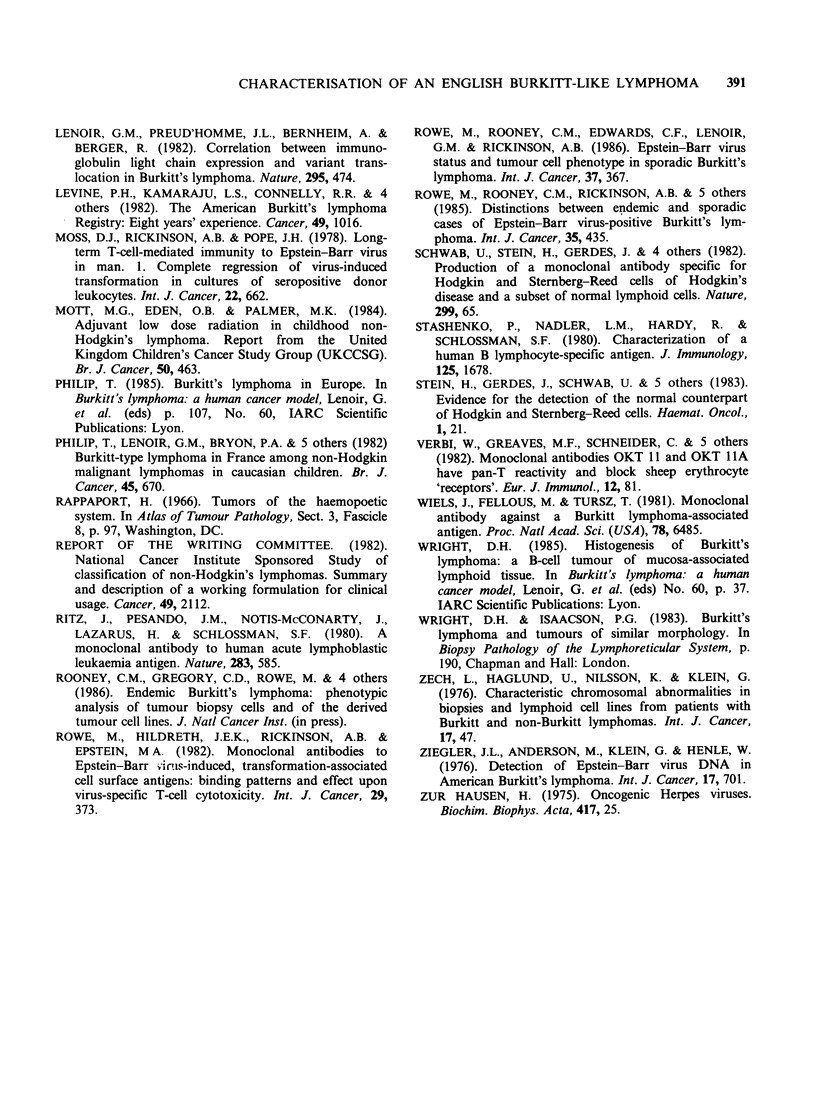# Burkitt-like lymphoma in an English child: characterisation of tumour biopsy cells and of the derived tumour cell line.

**DOI:** 10.1038/bjc.1986.188

**Published:** 1986-09

**Authors:** S. Finerty, M. Rowe, P. J. Berry, D. L. Ranson, M. G. Mott, C. D. Gregory, A. B. Rickinson

## Abstract

**Images:**


*Br. J. Cancer* (1986), **54**, 385–391

### Burkitt-like lymphoma in an English child: Characterisation of tumour biopsy cells and of the derived tumour cell line

S. Finerty[1], M. Rowe[2], P.J. Berry[3], D.L. Ranson[1], M.G. Mott[3], C.D. Gregory[2] & A.B. Rickinson[2]

[1]*Department of Pathology, University of Bristol Medical School, University Walk, Bristol BS8 1TD;* [2]*Department of Cancer Studies, University of Birmingham Medical School, Birmingham B15 2TJ; and* [3]*Bristol Royal Hospital for Sick Children, St. Michael's Hill, Bristol BS2 8BJ, UK.*

**Summary** An eight year old English boy presented with an abdominal undifferentiated 'Burkitt-like' lymphoma. Lymphoma cells from ascitic fluid were cultured on a human embryo fibroblast feeder layer and, after a short lag period, a cell line (DH-BL) was established which, like the original tumour, was both negative for the Epstein–Barr nuclear antigen (EBNA) and expressed a monoclonal pattern of surface immunoglobulin ($\alpha\lambda$). DH-BL also possessed the Burkitt-related 8:14 chromosome translocation in all metaphases analysed; no other chromosomal abnormalities were present. The cell surface phenotype of the original biopsy cells and the cultured tumour cells in early passage were investigated using a panel of monoclonal antibodies to B lineage-associated antigens. These antibodies had recently been used to characterise African 'endemic' Burkitt's lymphoma (BL) biopsy cells and their derived cell lines. The cell surface phenotype of this English EBNA negative Burkitt-like lymphoma biopsy was indistinguishable from that previously shown by biopsies of EBNA positive endemic BLs. It therefore appears that both the endemic and sporadic forms of BL, as illustrated by this case, may be derived from the same subset of progenitor cells.

When Burkitt's lymphoma (BL) was first described it was believed to be confined to certain parts of equatorial Africa and New Guinea coincident with holoendemic malarial infection. However, sporadic cases of the disease indistinguishable from BL on histological grounds began to be reported from countries outside these endemic areas (Burkitt, 1967). In addition to the histological similarities, both endemic and sporadic cases of BL display the same characteristic chromosomal translocation involving the long arm of chromosome 8 and a region of chromosome 14 or 2 or 22 near the relevant immunoglobulin gene locus (Lenoir *et al.*, 1982). However, there are significant differences between the two geographically separate forms of the disease. Firstly, they show different anatomical distributions. Endemic BL tends to involve the jaw and gonads whereas sporadic BL tends to involve the gastrointestinal tract, bone marrow or peripheral lymph nodes (Wright & Isaacson, 1983). Secondly, the Epstein-Barr (EB) virus is present in the malignant cells in at least 96% of endemic BL cases but in only a minority (15–20%) of sporadic BL cases (Ziegler *et al.*, 1976).

Debate continues as to whether BL should be considered as a single clinical entity, or should be classified into separate endemic and sporadic forms, or alternatively, into separate EB virus genome-positve and -negative forms (zur Hausen, 1975; de Thé *et al.*, 1978). In this context Rowe *et al.* (1985) have compared a number of EB virus-positive BL lines from both endemic and sporadic tumours for cell surface phenotype as defined by the cells' reactivity with a panel of monoclonal antibodies against B cell associated antigens. Cell lines derived from the two forms of virus-associated BL showed different ranges of phenotype and different growth patterns even within the first few *in vitro* passages. This suggested that the two forms of the tumour may be derived from distinct subsets of B cells *in vivo*, but the question could not be unequivocally resolved in the absence of data on fresh biopsy cells.

It was clearly important to extend this type of approach, analysing both tumour biopsy cells and the derived cell line, to cases of sporadic BL which were negative for the EB virus genome. The recent diagnosis of an EB virus-negative Burkitt-like lymphoma presenting in an English child provided the opportunity to characterise both the biopsy cells and the subsequent cell line from this tumour with the same panel of monoclonal antibody markers as used in the earlier work.

### Materials and methods

### *Clinical history*

The patient, DH, was an English male aged 8 years. Two weeks before admission he developed

Correspondence: S. Finerty.
Received 20 March 1986; and in revised form, 19 May 1986.

© The Macmillan Press Ltd., 1986

spasms of backache and ten days before admission complained of intermittent abdominal and limb pains. There was no significant past history or family history.

On examination he was found to have a palpable suprapubic mass 11 cm x 9 cm, arising from the pelvis. There was no palpable lymphadenopathy or hepatosplenomegaly. Ultrasound confirmed that the mass was solid in nature and showed dilated collecting systems of both kidneys, indicating mild ureteric obstruction. An exploratory laparotomy and biopsy were undertaken and imprints of the tumour showed lymphoblasts of characteristic L3 morphology (Bennett *et al.*, 1976). Bone marrow aspirate, trephine biopsy and lumbar puncture for cerebral spinal fluid cytology, showed no evidence of disease elsewhere.

The patient was treated according to a modification of the UKCCSG NHL protocol (Mott *et al.*, 1984). There was dramatic regression of the disease within hours of initiation of the treatment. Since that time, a period of 27 months, he has remained well with no evidence of recurrent disease.

The following specimens were obtained from the patient before therapy was commenced: heparinized peripheral blood ($10\,U\,ml^{-1}$) and ascites fluid collected in sterile tissue culture medium. The patient's plasma was found to be negative for IgG antibodies to EB VCA when tested by direct immunofluorescence (Henle & Henle, 1966).

### *Cell culture*

All cultures were carried out in RPMI 1640 medium supplemented with 2 mM glutamine, pencillin ($100\,IU\,ml^{-1}$), streptomycin ($100\,\mu g\,ml^{-1}$) and 10% v/v foetal calf serum (FCS) (Sera Lab., Crawley Down, Sussex, UK).

(*a*) *Establishment of lymphoma-derived cell line* (*DH-BL*) Viable mononuclear cells were isolated from the ascites sample by centrifugation over Ficoll-Hypaque by standard techniques. Aliquots of tumour cells were immediately cryopreserved in liquid nitrogen. Fresh tumour cells were cultured on human embryo fibroblasts in 2 ml Linbro plates at $10^6$ tumour cells per well as described fully elsewhere (Rooney *et al.*, 1986). The cultures were fed by replacing half the medium twice weekly. When growth commenced, the cells were initially subcultured onto fresh feeder cells and then into plastic tissue culture flasks.

(*b*) *Establishment of lymphoblastoid cell line* (*DH-LCL*) Mononuclear cells were separated from the whole blood by the method of Böyum (1968). Normal B cells within this population were infected with the B95-8 strain of EB virus exactly as in earlier work (Moss *et al.*, 1978; Finerty *et al.*, 1982) to give an EB virus-transformed B-lymphoblastoid cell line (LCL) of non-malignant origin. The LCL was maintained by replacing half the medium twice weekly.

### *Immunofluorescence tests*

*1. EB nuclear antigen* (*EBNA*) Cell smears were fixed in methanol:acetone (1:2) for 5 min at −20°C and stored at −20°C until required. The slides were stained for EBNA as described elsewhere (Crawford *et al.*, 1978).

*2. Surface and cytoplasmic immunoglobulin* Cells were analysed for surface and cytoplasmic immunoglobulin by standard direct immunofluorescence as previously described (Finerty *et al.*, 1982). Biopsy cells were also analysed for surface immunoglobulin by the method of Gregory *et al.* (1985).

*3. Surface phenotyping using monoclonal antibodies* The expression of various antigens at the cell surface was examined by indirect immunofluorescence using a panel of monoclonal antibodies referred to in Table I. Monoclonal antibodies MHM6, AC2, Ki-1, Ki-24 and 38.13 were used at dilutions of 1:100 to 1:500 of ascitic fluid preparations. The antibodies B1, J5 and OKT11 were obtained commercially and used as recommended by the suppliers. The FITC-conjugated second-step polyclonal antisera employed were a 1:100 dilution of goat anti-mouse IgG (Sigma, London) or, for the 38.13 rat monoclonal antibody, a 1:20 dilution of goat anti-rat IgM (Nordic Immunological Laboratories Ltd., Maidenhead), using as the diluent phosphate buffered saline (PBS) containing 10% EB virus antibody negative normal human serum and 10% normal goat serum.

The surface phenotyping was carried out as previously described: (a) on biopsy cells as described by Gregory *et al.*, (1985) and (b) on early *in vitro* passages of the cell lines under test as described by Rowe *et al.* (1985).

### *Chromosomal analysis*

Chromosome spreads were prepared and G-banded following the method of Autio and Schröder (1982). Briefly, desiccated chromosome slides were incubated at 60°C in 2 x SSC overnight, treated with 0.1% trypsin (Gibco Ltd., Paisley, Scotland) in PBS for 20 sec and stained in 2% giemsa (Gurr, BDH Chemicals Ltd., Poole, UK). Karyotypes were determined by the analysis of 20 metaphase spreads.

**Table I** Monoclonal antibodies used for cell surface phenotyping.

| *Monoclonal antibody* | *Specificity* | *Source* | *Reference* |
|---|---|---|---|
| B1 | Pan-B cell-associated antigen (35,000 mol. wt) | Coulter Clone | Stashenko *et al.* (1980) |
| J5 | cALLA (100,000 mol. wt) | Coulter Clone | Ritz *et al.* (1980) |
| 38.13 | BL cell-associated antigen (glycolipid) | J. Wiels | Wiels *et al.* (1981) |
| Ki-24 | Sternberg-Reed cell-associated antigen | H. Stein | Stein *et al.* (1983) |
| MHM6 | B-lymphoblastoid cell-associated antigen (45,000 mol. wt) | M. Rowe | Rowe *et al.* (1982) |
| AC2 | Lymphoblastoid cell-associated antigen (80,000 mol. wt) | M. Rowe | Rowe *et al.* (1982) |
| Ki-1 | Sternberg-Reed cell-associated antigen (110,000 mol. wt) | H. Stein | Schwab *et al.* (1982) |
| OKT11 | Pan-T cell-associated antigen (50,000 mol. wt) | Ortho Diagnostics | Verbi *et al.* (1982) |

### Results

### *Histopathology of the tumour*

Paraffin sections showed a diffuse non-Hodgkin's lymphoma composed of cells with scanty pyroninophilic cytoplasm and small non-cleaved nuclei approximating in size to those of adjacent histiocytes. Each nucleus contained two or more prominent nucleoli and had coarsely clumped chromatin. The mitotic rate was high and there was a conspicuous starry-sky pattern associated with the presence of many macrophages. In these respects the histological picture resembled that of endemic BL – although closer examination revealed some subtle differences. Thus the tumour cells showed more nuclear pleomorphism than is typical of endemic tumours and cytoplasmic lipid vacuoles, associated with endemic BL cells, were not seen by electronmicroscopy. The lymphoma was classified as Malignant Lymphoma, Small Noncleaved Type according to the Working Formulation of non-Hodgkin's Lymphomas (1982) or Undifferentiated Lymphoma according to the classification proposed by Rappaport (1966) (Figure 1).

Although the biopsy was small it contained intestinal smooth muscle on its outer aspect indicating that the lymphoma arose in the gastrointestinal tract.

**Figure 1** Histopathology of tumour showing lymphoma cells with scanty cytoplasm and small non-cleaved nuclei with prominent nucleoli. Many mitotic figures are present (arrows). One μm plastic section. H&E (×625).

### *Establishment of lymphoma-derived cell line (DH-BL) and of EB virus-transformed lymphoblastoid cell line (DH-LCL)*

In the case of the cultured biopsy cells, after a month of remaining apparently dormant on the human fibroblast feeder layer the lymphoma cells then began to proliferate and were subcultured into plastic tissue culture flasks. The appearance of the line was that of small cells with a slightly irregular outline, growing mainly as a single cell suspension but with some small loose clumps apparent even in the first few passages; clumping became slightly more pronounced after 6 months of continuous culture (Figure 2a).

EB virus-infected cultures of peripheral blood mononuclear cells gave rise to tight clumps of larger lymphoblastoid cells which could be subcultured within two weeks of infection to establish the LCL (Figure 2b).

### *Characterisation of DH biopsy cells, DH-BL and DH-LCL*

As shown in Table II both the tumour biopsy cells

**Figure 2** (*a*) Appearance in tissue culture of cell line derived from DH tumour cells – DH-BL – showing cells growing as single cells and small loose clumps. (*b*) Appearance in tissue culture of DH EB virus-transformed lymphoblastoid cell line – DH-LCL – showing cells growing in large tight clumps. Phase contrast photomicrographs.

and the tumour derived cell line, DH-BL, were EBNA negative and displayed the same monotypic $\alpha\lambda$ pattern of surface immunoglobulin expression. The tumour cell line also displayed the t(8:14) (q24q32) translocation (Figure 3) which is characteristic of BL (Zech *et al.*, 1976). In contrast DH-LCL was EBNA positive, polyclonal for both surface and cytoplasmic immunoglobulin and displayed a normal diploid karyotype.

These same cell populations were also examined for expression of B cell-associated markers as defined by the monoclonal antibodies listed in Table I. The results of repeated tests are summarised in Table III. The biopsy cell population was found to consist of 20% infiltrating T cells as identified with binding to the antibody OKT11 which recognises a pan T cell antigen. The percentages of fluorescent positive biopsy cells in Table III were therefore corrected to account only for the B cell fraction identified by binding to thc pan B cell antigen (recognised by the antibody B1). As expected, the cell populations from both DH-BL and DH-LCL were exclusively B cells as they gave 100% binding with the antibody B1. Both the tumour biopsy and the derived DH-BL cell line expressed two markers – the common acute lymphoblastic leukaemia antigen cALLA, defined by J5 staining, and the BL-associated antigen BLA, defined by 38.13 staining. These two markers have also been consistently found on all EB virus-positive BL cell lines studied in early passage (Rowe *et al.*, 1985). In addition the DH-BL cell line even when tested in early passage expressed the Ki-24 antigen on a minority of the cells but did not stain for any of the other 'lymphoblastoid' antigens defined by the antibodies MHM6, AC2 and Ki-1 (see below). DH-BL proved to be essentially stable on continued passaged (from observations over a period of six months) and was clearly distinguishable from that of DH-LCL. The latter cell line selectively bound Ki-24, MHM6, AC2 and Ki-1 antibodies exactly as described for all other LCLs thus analysed (Rowe *et al.*, 1985).

### Discussion

The present case report concerns an abdominal lymphoma in an English child which on clinical, histopathological and cytogenetic grounds clearly satisfies the criteria for 'sporadic BL' as employed by other groups (Levine *et al.*, 1982; Philip *et al.*, 1982). In particular, many features of the histopathology resembled those seen with the endemic disease and the tumour showed a t(8:14) chromosomal translocation, the commonest of three specific translocations consistently observed in association with BL (Berger & Bernheim, 1985). Like most sporadic BLs in Western Europe (Philip,

**Table II** Characteristics of DH biopsy, DH-BL and DH-LCL cells.

| *Cells* | *Cytoplasmic Ig* | *Surface Ig* | *EBNA* | *Karyotype* |
|---|---|---|---|---|
| DH Biopsy | NT[a] | Monoclonal $\alpha\lambda$ | Negative | NT |
| DH-BL | Negative | Monoclonal $\alpha\lambda$ | Negative | 46, XY, t(8:14)(q24q32) |
| DH-LCL | Polyclonal | Polyclonal | Positive | 46, XY |

[a]NT – not tested.

**Figure 3** Karyotype of metaphase chromosomes obtained from DH-BL cell line showing 8q− (small arrow) and 14q+ (large arrow).

**Table III** Comparison of monoclonal antibody binding of DH biopsy, DH-BL and DH-LCL.

| *Cells* | *In vitro growth pattern* | *Monoclonal antibody binding*[a] | | | | | | |
|---|---|---|---|---|---|---|---|---|
| | | *B1* | *J5* | *38.13* | *Ki-24* | *MHM6* | *AC2* | *Ki-1* |
| DH biopsy[b] | — | 100<br>+++ | 60<br>+ | 95<br>+++ | 0 | 0 | 0 | 0 |
| DH-BL | Single cells and small clumps | 100<br>+++ | 78<br>++ | 90<br>+++ | 25<br>+ | 0 | 0 | 0 |
| DH-LCL | Large clumps | 100<br>+++ | 0 | 0 | 74<br>++ | 79<br>++ | 80<br>+++ | 56<br>++ |

[a]Antibody binding was expressed as the percentage of fluorescent positive cells with an indication of the relative intensity of the staining. +++ = bright; ++ = moderate; + = weak. [b]Biopsy results were expressed as the percentage of the B cell population binding the monoclonal antibodies (see text).

1985), this tumour was EB virus-negative and indeed arose in a child whose serological status strongly suggested no previous EB virus infection. The tumour was somewhat unusual, although not unique amongst cases of sporadic BL (Rowe *et al.*, 1985) in expressing surface immunoglobulin of class $\alpha$ rather than class $\mu$.

One of the key issues in questioning the relationship between endemic and sporadic BL is the identity of the target cell involved in each case. Previous work has established a panel of monoclonal antibodies, against B cell-associated surface antigens, which has proved particularly useful in defining the various surface phenotypes displayed by BL cells in culture (Rowe *et al.*, 1985). These antibodies have recently been used to characterise endemic BL biopsy cells and their derived cell lines (Rooney *et al.*, 1986) and the present report represents the first extension of this approach to a case of sporadic BL. The results are interesting in that the cell surface phenotype of the DH-BL biopsy (Table III) was indistinguishable from that shown by all biopsies of EB virus-positive endemic BLs so far examined (Rooney *et al.*, 1986).

Related work with EB virus-positive BL has revealed a tendency for many of the derived cell lines to progress towards a more 'lymphoblastoid' phenotype with continued passage *in vitro*, without loss of the cytogenetic markers indicative of malignant origin (Rowe *et al.*, 1985; Rooney *et al.*, 1986).

The present results with an EB virus-negative BL cell line are contrary to this general pattern, since continual culture of the DH-BL line was only associated with acquisition of a weak reactivity with the antibody Ki-24 but no further progression towards the 'lymphoblastoid' phenotype. One possibility is that the more dramatic examples of phenotypic progression *in vitro* represent the influence of a resident EB virus genome upon an otherwise stable BL cell phenotype. This view is indeed supported by the recent observation that several other established EB virus-negative BL cell lines show the same phenotype as described here for DH-BL cells (Rowe *et al.*, 1986).

The overall inference from these studies is that, despite minor differences in histopathological appearance between the DH tumour and a typical case of endemic BL, the tumour cell surface phenotypes *in vivo* were indistinguishable (at least by the panel of markers employed). Although much more work will be required, this certainly raises the possibility that both the endemic and sporadic forms of BL are derived from a cALLA-positive BLA-positive subset of progenitor cells. Such a normal subset has not yet been unequivocally defined but it certainly becomes important to search for such normal cells in lymphoid tissue, particularly in mucosa-associated lymphoid tissue where BL is considered to originate (Wright, 1985). Furthermore it is important to examine the precise position of such cells within the B cell lineage.

This work was supported by funds from the Cancer Research Campaign, London, from the Medical Research Council, and from the Cancer and Leukaemia in Childhood Trust (CLIC). The authors are most grateful to Miss Jill Sweet for invaluable technical help.

### References

AUTIO, K. & SCHRÖDER, J. (1982). Chromosome break points in clonal and non clonal chromosome changes in human chronic lymphocytic leukaemia. *Hereditas*, **97**, 221.

BENNETT, J.M., CATOVSKY, D., DANIEL, M. & 4 others (1976). Proposals for the classification of the acute leukaemias. *Br. J. Haematology*, **33**, 451.

BERGER, R. & BERNHEIM, A. (1985). Cytogenetics of Burkitt's lymphoma-leukaemia: a review. In *Burkitt's lymphoma: a human cancer model*, Lenoir G. *et al.* (eds) p. 65, No. 60, IARC Scientific Publications: Lyon.

BÖYUM, A. (1968). Separation of leucocytes from blood and bone marrow. *Scand. J. Clin. Lab. Invest.*, **21**, Suppl. 97, 77.

BURKITT, D. (1967). Burkitt's lymphoma outside the known endemic areas of Africa and New Guinea. *Int. J. Cancer*, **2**, 562.

CRAWFORD, D.H., RICKINSON, A.B., FINERTY, S. & EPSTEIN, M.A. (1978). Epstein–Barr (EB) virus genome-containing, EB nuclear antigen-negative B lymphocyte populations in blood in acute infectious mononucleosis. *J. Gen. Virol.*, **38**, 449.

DE THÉ, G., GESER, A., DAY, N.E., TUKEI, P.M., WILLIAMS, E.H., BERI, D.P., SMITH, P.G., DEAN, A.G., BORNKAMM, G.W., FEORINO, P. & HENLE, W. (1978). Epidemiological evidence for causal relationship between Epstein–Barr virus and Burkitt's lymphoma from Ugandan prospective study. *Nature*, **274**, 756.

FINERTY, S., RICKINSON, A.B., EPSTEIN, M.A. & PLATTS-MILLS, T.A.E. (1982). Interaction of Epstein–Barr virus with leukaemic B cells *in vitro*. II. Cell line establishment from prolymphocytic leukaemia and from Waldenström's macroglobulinaemia. *Int. J. Cancer*, **30**, 1.

GREGORY, C.D., LEE, M., REES, G.B., SCOTT, I.V., SHAH, L.P. & GOLDING, P.R. (1985). Natural killer cells in normal pregnancy: analysis using monoclonal antibodies and single-cell cytotoxicity assays. *Clin. Exp. Immunol.*, **62**, 121.

HENLE, G. & HENLE, W. (1966). Immunofluorescence in cells derived from Burkitt's lymphoma. *J. Bacteriol.*, **91**, 1248.

LENOIR, G.M., PREUD'HOMME, J.L., BERNHEIM, A. & BERGER, R. (1982). Correlation between immunoglobulin light chain expression and variant translocation in Burkitt's lymphoma. *Nature*, **295**, 474.

LEVINE, P.H., KAMARAJU, L.S., CONNELLY, R.R. & 4 others (1982). The American Burkitt's lymphoma Registry: Eight years' experience. *Cancer*, **49**, 1016.

MOSS, D.J., RICKINSON, A.B. & POPE, J.H. (1978). Long-term T-cell-mediated immunity to Epstein–Barr virus in man. 1. Complete regression of virus-induced transformation in cultures of seropositive donor leukocytes. *Int. J. Cancer*, **22**, 662.

MOTT, M.G., EDEN, O.B. & PALMER, M.K. (1984). Adjuvant low dose radiation in childhood non-Hodgkin's lymphoma. Report from the United Kingdom Children's Cancer Study Group (UKCCSG). *Br. J. Cancer*, **50**, 463.

PHILIP, T. (1985). Burkitt's lymphoma in Europe. In *Burkitt's lymphoma: a human cancer model*, Lenoir, G. *et al.* (eds) p. 107, No. 60, IARC Scientific Publications: Lyon.

PHILIP, T., LENOIR, G.M., BRYON, P.A. & 5 others (1982) Burkitt-type lymphoma in France among non-Hodgkin malignant lymphomas in caucasian children. *Br. J. Cancer*, **45**, 670.

RAPPAPORT, H. (1966). Tumors of the haemopoetic system. In *Atlas of Tumour Pathology*, Sect. 3, Fascicle 8, p. 97, Washington, DC.

REPORT OF THE WRITING COMMITTEE. (1982). National Cancer Institute Sponsored Study of classification of non-Hodgkin's lymphomas. Summary and description of a working formulation for clinical usage. *Cancer*, **49**, 2112.

RITZ, J., PESANDO, J.M., NOTIS-McCONARTY, J., LAZARUS, H. & SCHLOSSMAN, S.F. (1980). A monoclonal antibody to human acute lymphoblastic leukaemia antigen. *Nature*, **283**, 585.

ROONEY, C.M., GREGORY, C.D., ROWE, M. & 4 others (1986). Endemic Burkitt's lymphoma: phenotypic analysis of tumour biopsy cells and of the derived tumour cell lines. *J. Natl Cancer Inst.* (in press).

ROWE, M., HILDRETH, J.E.K., RICKINSON, A.B. & EPSTEIN, M.A. (1982). Monoclonal antibodies to Epstein–Barr virus-induced, transformation-associated cell surface antigens: binding patterns and effect upon virus-specific T-cell cytotoxicity. *Int. J. Cancer*, **29**, 373.

ROWE, M., ROONEY, C.M., EDWARDS, C.F., LENOIR, G.M. & RICKINSON, A.B. (1986). Epstein–Barr virus status and tumour cell phenotype in sporadic Burkitt's lymphoma. *Int. J. Cancer*, **37**, 367.

ROWE, M., ROONEY, C.M., RICKINSON, A.B. & 5 others (1985). Distinctions between endemic and sporadic cases of Epstein–Barr virus-positive Burkitt's lymphoma. *Int. J. Cancer*, **35**, 435.

SCHWAB, U., STEIN, H., GERDES, J. & 4 others (1982). Production of a monoclonal antibody specific for Hodgkin and Sternberg–Reed cells of Hodgkin's disease and a subset of normal lymphoid cells. *Nature*, **299**, 65.

STASHENKO, P., NADLER, L.M., HARDY, R. & SCHLOSSMAN, S.F. (1980). Characterization of a human B lymphocyte-specific antigen. *J. Immunology*, **125**, 1678.

STEIN, H., GERDES, J., SCHWAB, U. & 5 others (1983). Evidence for the detection of the normal counterpart of Hodgkin and Sternberg–Reed cells. *Haemat. Oncol.*, **1**, 21.

VERBI, W., GREAVES, M.F., SCHNEIDER, C. & 5 others (1982). Monoclonal antibodies OKT 11 and OKT 11A have pan-T reactivity and block sheep erythrocyte 'receptors'. *Eur. J. Immunol.*, **12**, 81.

WIELS, J., FELLOUS, M. & TURSZ, T. (1981). Monoclonal antibody against a Burkitt lymphoma-associated antigen. *Proc. Natl Acad. Sci.* (*USA*), **78**, 6485.

WRIGHT, D.H. (1985). Histogenesis of Burkitt's lymphoma: a B-cell tumour of mucosa-associated lymphoid tissue. In *Burkitt's lymphoma: a human cancer model*, Lenoir, G. *et al.* (eds) No. 60, p. 37. IARC Scientific Publications: Lyon.

WRIGHT, D.H. & ISAACSON, P.G. (1983). Burkitt's lymphoma and tumours of similar morphology. In *Biopsy Pathology of the Lymphoreticular System*, p. 190, Chapman and Hall: London.

ZECH, L., HAGLUND, U., NILSSON, K. & KLEIN, G. (1976). Characteristic chromosomal abnormalities in biopsies and lymphoid cell lines from patients with Burkitt and non-Burkitt lymphomas. *Int. J. Cancer*, **17**, 47.

ZIEGLER, J.L., ANDERSON, M., KLEIN, G. & HENLE, W. (1976). Detection of Epstein–Barr virus DNA in American Burkitt's lymphoma. *Int. J. Cancer*, **17**, 701.

ZUR HAUSEN, H. (1975). Oncogenic Herpes viruses. *Biochim. Biophys. Acta*, **417**, 25.